# Quality of life in advanced cancer patients: the impact of sociodemographic and medical characteristics

**DOI:** 10.1054/bjoc.2001.2116

**Published:** 2001-11

**Authors:** M S Jordhøy, P Fayers, J H Loge, T Saltnes, M Ahlner-Elmqvist, S Kaasa

**Affiliations:** Unit of Applied Clinical Research, University Hospital of Trondheim, NTNU, 5. etg., Kreftbygget, Trondheim, N-7006, Norway; Unit of Applied Clinical Research, NTNU, Trondheim, Norway; Unit of Applied Clinical Research, NTNU, Trondheim; Department of Oto-Rhino-Laryngology, Malmö University Hospital, Malmö, S-20502, Sweden; Palliative Medicine Unit, Department of Radiotherapy and Oncology, University Hospital of Trondheim and Unit of Applied Clinical Research, NTNU, 5 etg. Kreftbygget, RiT, Trondheim, N-7006, Norway; Department of Public Health, Aberdeen Medical School, Aberdeen, AB25 2ZD, UK; Department of Behavioral Sciences in Medicine, University of Oslo, p.b. 1111 Blindern, Oslo, 0317

**Keywords:** palliative care, cancer, quality of life, predictive factors

## Abstract

Population-based surveys have shown that health-related quality of life (HRQL) is influenced by patients' characteristics such as age, gender, living situation and diagnoses. The present study explores the impact of such factors on the HRQL of severely ill cancer patients. The study sample included 395 cancer patients who participated in a cluster randomised trial of palliative care. Median survival was 13 weeks. HRQL assessments (using the EORTC QLQ-C30 questionnaire) were compared among subgroups of relevant patients' characteristics (ANOVA), and the significance of individual covariates was explored by multivariate linear regression. Most EORTC QLQ-C30 scores showed minor differences between genders. Higher age was associated with less sleeping disturbance, less pain and better emotional functioning. No positive impact of living with a partner was found. Performance status and/or time from assessment to death were significantly associated with most functioning and symptom scores. We concluded that although the overall impact of sociodemographic characteristics may seem less important to HRQL scores among advanced cancer patients than in general populations, age and gender should be allowed for. Performance status and closeness to death also need to be reported.   http://www.bjcancer.com © 2001 Cancer Research Campaign


*British Journal of Cancer* (2001) **85**(10), 1478–1485
© 2001 Cancer Research Campaign
doi: 10.1054/ bjoc.2001.2116, available online at http://www.idealibrary.com on IDEAL

http://www.bjcancer.com

### Quality of life in advanced cancer patients: the impact of sociodemographic and medical characteristics

**MS Jordhøy[1], P Fayers[2], JH Loge[3], T Saltnes[1], M Ahlner-Elmqvist[4] and S Kaasa[5]**

Unit of Applied Clinical Research, NTNU, 5. etg., Kreftbygget, University Hospital of Trondheim, N-7006 Trondheim, Norway; [2]Unit of Applied Clinical Research, NTNU, Trondheim, Norway and Department of Public Health, Aberdeen Medical School, Aberdeen AB25 2ZD, UK; [3]Unit of Applied Clinical Research, NTNU, Trondheim, and Department of Behavioral Sciences in Medicine, University of Oslo, p.b. 1111 Blindern, 0317 Oslo; [4]Department of Oto-Rhino-Laryngology, Malmö University Hospital, S-20502 Malmö Sweden; [5]Palliative Medicine Unit, Department of Radiotherapy and Oncology, University Hospital of Trondheim and Unit of Applied Clinical Research, NTNU, 5 etg. Kreftbygget, RiT, N-7006 Trondheim, Norway

**Summary** Population-based surveys have shown that health-related quality of life (HRQL) is influenced by patients' characteristics such as age, gender, living situation and diagnoses. The present study explores the impact of such factors on the HRQL of severely ill cancer patients. The study sample included 395 cancer patients who participated in a cluster randomised trial of palliative care. Median survival was 13 weeks. HRQL assessments (using the EORTC QLQ-C30 questionnaire) were compared among subgroups of relevant patients' characteristics (ANOVA), and the significance of individual covariates was explored by multivariate linear regression. Most EORTC QLQ-C30 scores showed minor differences between genders. Higher age was associated with less sleeping disturbance, less pain and better emotional functioning. No positive impact of living with a partner was found. Performance status and/or time from assessment to death were significantly associated with most functioning and symptom scores. We concluded that although the overall impact of sociodemographic characteristics may seem less important to HRQL scores among advanced cancer patients than in general populations, age and gender should be allowed for. Performance status and closeness to death also need to be reported. © 2001 Cancer Research Campaign http://www.bjcancer.com

**Keywords**: palliative care; cancer; quality of life; predictive factors

The primary goal of palliative cancer care is to improve, or maintain, patients' health-related quality of life (HRQL). Subjective outcome measures including physical, psychological and social well-being are therefore frequently used to assess the effect of palliative care interventions (Smeenk et al, 1998; Salisbury et al, 1999). Population-based studies, however, have shown that patient characteristics such as age, gender, marital status and diagnoses may have a profound impact on HRQL-ratings (Brazier et al, 1992; Jenkinson et al, 1993; Klee et al, 1997; Hjermstad et al, 1998a, 1998b). Hence, HRQL outcomes may be confounded by such factors, which consequently need consideration in study design and statistical analyses as well as in the comparison of results among different trials. This is particularly relevant in palliative care research, where the target population is generally very heterogeneous, and the patient selection may vary substantially from one setting to another (Hearn and Higginson, 1998; Wilkinson, 1999).

However, HRQL may change with time and circumstances, and what has been found to influence the HRQL in population-based surveys, may not be valid among patients who are severely ill (Cohen and Mount, 1992). The main purpose of this study was therefore to explore the impact of sociodemographic and medical characteristics on the HRQL of patients with incurable cancer and short survival expectancy. The HRQL assessments were made using the EORTC QLQ-C30 questionnaire (Aaronson et al, 1993).

*Received 12 March 2001*
*Revised 25 July 2001*
*Accepted 30 July 2001*

*Correspondence to:* MS Jordhøy

According to normative data (Hjermstad et al, 1998a), older people report worse functioning and more symptoms than those who are younger. This may mainly be related to comorbidity (Moum, 1992; Hjermstad et al, 1998b; Michelson et al, 2000), and as HRQL has been found to vary with clinical criteria such as performance status and prognoses (Osoba et al, 1994; King, 1996), our hypothesis was that the level of functioning impairment and symptom level among our patients would depend on disease-related factors rather than age. One exception was assumed. Older persons with cancer have been found to manifest fewer and less severe psychosocial problems (Mor et al, 1994), thus, we postulated that emotional functioning would be better among the oldest. Overall, having higher education, being male or being married rather than living alone have been found to exert a positive influence on HRQL (Sullivan et al, 1994; Hjermstad et al, 1998a; Michelson et al, 2000). Similar findings were expected among advanced cancer patients. In particular, we found it reasonable to believe that having a partner would be important to their sense of well-being and emotional and social functioning.

### MATERIAL AND METHODS

### Design and study sample

The study sample originated from a cluster randomised trial comparing the service of the Palliative Medicine Unit at the University Hospital of Trondheim, Norway to conventional care. The design, recruitment and outcome measures have been thoroughly

The study was approved by the Ethical Review Committee at the Norwegian University of Science and Technology (NTNU), Trondheim, Norway.

described elsewhere (Jordhoy et al, 1999, 2000). Inclusion criteria were incurable, malignant disease, life expectancy between 2 and 9 months and age above 18. Completion of the baseline HRQL assessment, which included the EORTC QLQ-C30 questionnaire, was mandatory for trial entry (Jordhoy et al, 1999).

Between March 1995 and November 1997, 434 patients were entered onto trial, of whom 16 patients withdrew while 23 were still alive at the end of follow-up (2 years). A total of 395 patients died, and to be able to take time from assessment to death into account, only these patients were included in the present study sample. Median age was 70 years, 47% was female, and the majority was living with their spouse (Table 1). Education was recorded as number of years at school. 'Seven years' was the minimum educational requirement up to 1968. After this, the public compulsory education was increased by 2 more years. More than 13 years corresponds to studies at the university level (Table 1). Neither former nor present type of occupation was recorded. At trial entry, only 10 patients (2.5%) were still working. Thus, exploring any impact of working status would not be meaningful. Cancers of the digestive system were the most frequent diagnoses,

**Table 1** Patients' sociodemographic and medical characteristics

| | All patients (*n* = 395) | |
|---|---|---|
| | ***n*** | **%** |
| Gender | | |
| females | 184 | 46.6 |
| males | 211 | 53.4 |
| Age (years) | | |
| median (min–max) | 69.5 | (37–93) |
| Married or cohabitating | | |
| yes | 251 | 63.5 |
| no | 144 | 36.5 |
| Living situation | | |
| alone | 127 | 32.2 |
| with spouse or partner +/– children | 249 | 63.0 |
| with others | 19 | 4.8 |
| Education | | |
| ≤ 7 years | 141 | 35.7 |
| 8–10 years | 140 | 35.4 |
| 11–12 years | 56 | 14.2 |
| ≥ 13 years | 58 | 14.7 |
| Cancer origin | | |
| Gastro-intestine | 168 | 42.5 |
| Lung | 48 | 12.2 |
| Breast and female genitals | 57 | 14.4 |
| Prostate and male genitals | 36 | 9.1 |
| Others | 86 | 21.8 |
| Weeks from diagnosis to inclusion-median (min-max) | 36 | (1–1469) |
| Karnofsky status | | |
| 100 | 43 | 10.9 |
| 90 | 93 | 23.5 |
| 80 | 98 | 24.8 |
| 70 | 79 | 20.0 |
| 60 | 52 | 13.2 |
| 50 | 23 | 5.8 |
| 40 | 7 | 1.8 |
| < 40 | 0 | 0.0 |
| Skeletal metastasis | 85 | 21.5 |
| Lung metastasis | 61 | 15.4 |
| Liver metastasis | 139 | 35.2 |
| Ongoing chemotherapy | 52 | 13.2 |
| Ongoing radiotherapy | 42 | 10.6 |

and 41% of the patients had a Karnofsky performance status of 70 or less (Table 1). Median survival was 13 weeks (range 0–125 weeks).

For the purpose of this study, EORTC QLQ-C30 scores obtained at trial entry were analysed. Due to death or withdrawal, the sample size decreased during the follow-up, hence the baseline scores were chosen to achieve the best statistical power. These scores were also considered to be the most appropriate for the analyses because some relevant medical characteristics (e.g. performance status and location of metastasis) were only recorded at enrolment.

### The EORTC QLQ-C30 questionnaire

The EORTC QLQ-C30 questionnaire (Aaronson et al, 1993; Fayers et al, 1995; Kaasa et al, 1995) includes a total of 30 items and is composed of scales that evaluate physical (5 items), emotional functioning (4 items), role (2 items), cognitive (2 items) and social (2 items) functioning as well as global health status (2 items). Higher mean scores on these scales represent better functioning. The questionnaire also comprises 3 symptom scales measuring nausea and vomiting (2 items), fatigue (3 items) and pain (2 items), and 6 single items assessing financial impact and various physical symptoms. Higher scores on these scales/items mean more symptoms. Before statistical analyses, the raw EORTC QLQ-C30 scores are linearly transformed to a 0–100 scale (Fayers et al, 1995). A mean change in scores of 5 to 10 has been found to represent 'a little' subjective change to the patients, while a change of 10 to 20 represents a moderate change (Osoba et al, 1998). Thus, differences of 10 points or more may be regarded as clinically significant.

Missing items were imputed by the method advocated by the EORTC QLQ research group; if at least half of the items from a scale were completed, the mean value for these items was imputed for those missing (Fayers et al, 1995). The overall proportion of missing items was 1.5%.

### Statistics

HRQL scores were compared between subgroups according to patients' sociodemographic and medical characteristics using Students' *t*-test and one-way analyses of variance (ANOVA). Post hoc, and for characteristics including more than 2 nominal categories, multiple comparisons with Bonferroni adjustment were used to identify differing subgroups, whereas for factors with ordered categories, that is, categories defined according to performance status, years of education, age and closeness to death, differences were tested for linear trends (equivalent to linear regression) (Altman 1991).

Thereafter, the impact of sociodemographic and medical factors on symptom and functioning scores was explored by multivariate linear regression, starting with models including all explanatory variables listed in Table 1, except marital status. The latter was closely linked to living situation and therefore not analysed. Living situation and performance status were dichotomised ('living with a spouse', yes/no; Karnofsky status > 70, yes/no), whereas for diagnoses and education, dummy variables were created. Age was included as a continuous measure. A backward stepwise approach was then used to build models excluding factors that did not contribute significantly to the HRQL scores. Finally, to explore if the non-eliminated factors maintained their predictive significance when closeness to death was allowed for, or if closeness to death itself was associated with the HRQL

© 2001 Cancer Research Campaign scores, time from assessment to death was added to the models, and the backward stepwise procedure was repeated.

The described statistical methods assume normal distribution of the data. For most of the multi-item EORTC QLQ-C30 scales this represented a reasonable approximation, and for the ease of interpretation, the results according to these methods are presented. However, non-parametric tests and ordered logistic regression were used to confirm all results, and any discrepancies are explicitly stated.

The regression modelling was done by Stata version 5 (Stata Corporation, 1997), otherwise SPSS version 9.0 (SPSS Inc, 1999) was used. The $P$ value for significance was set at $P = 0.01$ to provide some protection from multiple comparisons. For the multiple regression, time factors were treated as continuous variables, that is, the logarithms of time from diagnoses to assessment (Table 1) and of time from assessment to death were used.

### RESULTS

### HRQL according to sociodemographic factors, diagnoses and location of metastasis

Very few patients ($n = 33$) were below 50 years of age, thus age was categorised as in Table 2. Older age was significantly associated with better emotional and social functioning, less sleeping disturbance and a lower financial impact (Table 2). The oldest patients also tended to report more appetite loss, while most pain was found among the youngest. These differences, however, were not statistically significant.

The EORTC scores according to gender and living situation are shown in Table 3. Overall, men reported better functioning and lower level of symptoms, but the differences were minor and statistically significant only for physical functioning, fatigue and nausea/vomiting. The results of comparing those who were living with their spouse to those not doing so were similar to comparing those who were living with someone to those who were alone. In both cases, there was a significantly lower social functioning among the former group (mean 45 with spouse/partner versus 54 without). Otherwise, no statistically or clinically significant differences were found.

None of the EORTC QLQ-C30 scales/items differed significantly according to levels of education (data not shown). However, better emotional functioning (72 versus 65) was indicated among patients with university education compared to those having 7 years at school or less. The former group also had the lowest pain scores, whereas patients with a median level of education reported poorer general well-being than those with both highest and lowest level (35 versus 43 and 42).

ANOVA tests comparing subgroups according to diagnoses revealed significant differences for physical functioning, dyspnoea, diarrhoea and constipation ($P < 0.01$) (Table 3). Patients with gastro-intestinal cancer reported the best physical functioning, and significantly better than those having cancer of the breast and female genitals, and except for the latter group, patients with lung cancer reported significantly more dyspnoea than the others ($P < 0.01$, Bonferroni) (Table 3). Most diarrhoea and least constipation were reported among patients with gastrointestinal cancers. For these scores, however, post hoc tests failed to identify any subgroup differences within the defined level of significance.

Patients with skeletal metastasis had significantly lower physical functioning, more pain and constipation than the others (Table 3). Liver metastases were associated with lower pain and constipation scores whereas patients with metastasis to the lungs tended to report more dyspnoea ($P = 0.02$, ns) (Table 3). Overall, patients who were on chemotherapy reported worse functioning and more symptoms than those who were not, however, few differences were statistically significant (Table 3).

### HRQL scores according to closeness to death

To explore the association between the HRQL scores and survival, the patients were grouped according to time from assessment to death. The scores of those patients who completed the baseline

**Table 2** EORTC QLQ-C30 functioning and symptom scores according to age

| | All patients ($n$ = 395) | | < 50 years ($n$ = 33) | 50–59 years ($n$ = 59) | 60–69 years ($n$ = 110) | 70–79 years ($n$ = 135) | 80 > years ($n$ = 58) |
|---|---|---|---|---|---|---|---|
| | mean | (SD) | mean | mean | mean | mean | mean |
| Functioning scales | | | | | | | |
| Physical | 46 | (31) | 46 | 54 | 47 | 46 | 35 |
| Emotional[a] | 66 | (26) | 56 | 59 | 64 | 68 | 76 |
| Role | 32 | (31) | 24 | 32 | 29 | 34 | 33 |
| General well-being | 39 | (26) | 36 | 40 | 38 | 40 | 38 |
| Cognitive | 72 | (27) | 66 | 70 | 75 | 70 | 76 |
| Social[a] | 48 | (33) | 38 | 36 | 47 | 53 | 58 |
| Symptom scales | | | | | | | |
| Fatigue | 65 | (27) | 67 | 64 | 64 | 62 | 72 |
| Nausea/vomiting | 25 | (30) | 19 | 22 | 28 | 23 | 30 |
| Pain | 48 | (36) | 59 | 53 | 46 | 46 | 48 |
| Single items | | | | | | | |
| Diarrhoea | 26 | (34) | 23 | 26 | 27 | 28 | 22 |
| Dyspnoea | 39 | (36) | 46 | 45 | 42 | 37 | 33 |
| Appetite | 51 | (39) | 43 | 43 | 51 | 51 | 61 |
| Sleeping disturbance[a] | 40 | (36) | 59 | 45 | 39 | 34 | 40 |
| Constipation | 43 | (39) | 49 | 44 | 40 | 40 | 48 |
| Financial impact[a] | 16 | (27) | 28 | 19 | 17 | 14 | 9 |

[a]For these scales the differences in scores between age groups were statistically significant (ANOVA, $P = < 0.01$), and a significant linear trend across subgroups was found ($P < 0.01$).

 © 2001 Cancer Research Campaign

**Table 3** Functioning and symptoms scores according to sociodemographic and medical factors

| | Functioning scales[a] | | | | | | Symptom scales[b] | | | | Symptoms – single items[b] | | | | |
|---|---|---|---|---|---|---|---|---|---|---|---|---|---|---|---|
| | PF mean | EF mean | RF mean | QL mean | CF mean | SF mean | FA mean | NV mean | PA mean | DI mean | DY mean | AP mean | SL mean | CO mean | FI mean |
| Gender | | | | | | | | | | | | | | | |
| male | [c]52 | 68 | 34 | 41 | 75 | 51 | [c]61 | [c]21 | 47 | 23 | 40 | 48 | 39 | 40 | 15 |
| female | 39 | 63 | 29 | 36 | 69 | 45 | 69 | 29 | 49 | 30 | 38 | 54 | 41 | 46 | 18 |
| Living with a spouse | | | | | | | | | | | | | | | |
| no | 43 | 66 | 33 | 37 | 72 | [c]54 | 67 | 29 | 48 | 29 | 39 | 55 | 38 | 41 | 16 |
| yes | 47 | 66 | 31 | 40 | 72 | 45 | 63 | 22 | 48 | 24 | 40 | 48 | 41 | 43 | 16 |
| Cancer origin | | | | | | | | | | | | | | | |
| gastro-intestine | [c]52 | 69 | 35 | 40 | 75 | 51 | 65 | 25 | 47 | [c]33 | [c]34 | 52 | 38 | [c]34 | [c]11 |
| lungs | 36 | 59 | 25 | 35 | 63 | 45 | 70 | 26 | 47 | 21 | 64 | 53 | 48 | 48 | 22 |
| breast/female genitals | 33 | 58 | 26 | 35 | 67 | 40 | 70 | 29 | 50 | 23 | 52 | 51 | 42 | 52 | 25 |
| prostate/male genitals | 43 | 68 | 30 | 40 | 72 | 50 | 61 | 25 | 61 | 14 | 33 | 45 | 41 | 56 | 13 |
| other | 49 | 68 | 33 | 41 | 74 | 49 | 59 | 20 | 45 | 21 | 31 | 49 | 38 | 44 | 19 |
| Having skeletal metastasis | | | | | | | | | | | | | | | |
| no | [c]48 | 67 | 32 | 40 | 72 | 49 | 64 | 25 | [c]44 | 28 | 40 | 50 | 40 | [c]39 | 16 |
| yes | 36 | 62 | 29 | 36 | 71 | 45 | 67 | 25 | 64 | 19 | 38 | 51 | 39 | 56 | 18 |
| Having liver metastasis | | | | | | | | | | | | | | | |
| no | 43 | 66 | 30 | 37 | [c]69 | 47 | 65 | 26 | [c]52 | 24 | 41 | 50 | 41 | [c]47 | [c]19 |
| yes | 51 | 67 | 34 | 42 | 77 | 50 | 63 | 22 | 42 | 29 | 37 | 52 | 37 | 35 | 10 |
| Having lung metastasis | | | | | | | | | | | | | | | |
| no | 45 | 66 | 32 | 39 | 72 | 47 | 65 | 25 | 48 | 26 | 38 | 50 | 40 | [c]45 | 16 |
| yes | 49 | 67 | 31 | 38 | 72 | 55 | 61 | 22 | 47 | 24 | 49 | 51 | 38 | 31 | 15 |
| Having chemotherapy | | | | | | | | | | | | | | | |
| no | 47 | 67 | 33 | 39 | 73 | [c]50 | 63 | 24 | 47 | 26 | [c]37 | 50 | 39 | [c]40 | 16 |
| yes | 41 | 58 | 22 | 36 | 66 | 36 | 72 | 33 | 53 | 24 | 53 | 58 | 49 | 58 | 19 |
| Having radiotherapy | | | | | | | | | | | | | | | |
| no | 45 | 66 | 31 | 39 | 72 | 47 | 65 | 25 | 47 | 27 | 40 | 52 | 40 | [c]41 | 16 |
| yes | 49 | 69 | 35 | 40 | 75 | 57 | 61 | 23 | 54 | 20 | 33 | 41 | 42 | 59 | 18 |

[a]Functioning (PF = physical, EF = emotional, RF = role, QL = general well-being, CF = cognitive, SF = social): higher sores mean better functioning;
[b]symptom scales and single items (FA = fatigue, NV = nausea/vomiting, PA = pain, DI = diarrhea, DY = dyspnea, AP = appetite loss, SL = sleeping disturbance, CO = constipation, FI = financial impact): higher scores mean more symptoms.
[c]$P \leq 0.01$, ANOVA or Students' *t*-test for significance of differences between subgroups.

© 2001 Cancer Research Campaign questionnaire within 1, 2, 3, 4 and 5 months or more prior to death respectively were compared. Between these groups, significant differences ($P < 0.005$) were found on all functioning scales except social functioning ($P = 0.016$) as well as on appetite loss, dyspnoea, fatigue, nausea /vomiting, pain and constipation. For the same scales/items, social functioning included, there was also a significant linear trend across subgroups ($P < 0.002$) (Figure 1). Overall, the worse scores were obtained within the last month of life, whereas those patients who lived for 91–120 days or more reported the best functioning and least symptoms. These differences were also clinically significant, that is, varying from 11 (emotional functioning) to 38 points (appetite loss). No association between closeness to death and diarrhoea or sleeping disturbances was found.

### HRQL scores according to performance status

Performance status was measured using the Karnofsky index. For all functioning and symptom scores, the worse ratings were obtained from patients with the poorest status. The difference between subgroups (Karnofsky index 40 to 100) (Table 1) was statistically significant for all functioning scales except emotional functioning, as well as for fatigue, pain, appetite loss and constipation ($P < 0.002$). Emotional functioning included, these scores also showed a significant linear trend across subgroups (emotional functioning: $P = 0.006$; others: $P < 0.001$), that is, better performance status was associated with better scores and poorer status with worse scores (Figure 2). As can be noted, the scores on social, role and physical functioning as well as general well-being among patients with Karnofsky status of 40 were very low, indicating a ‘floor effect’, or a poor ability to discriminate between subgroups of such patients. Diarrhoea, sleeping disturbances and dyspnoea showed minor variations, whereas subgroup differences in nausea and vomiting scores were close to significant ($P = 0.011$) and showed a significant linear trend ($P = 0.001$) (Figure 2).

The difference in HRQL ratings between groups classified according to whether Karnofsky status was more than 70 or not, were also statistically significant ($P < 0.002$) for the same scales and items as above, and for nausea and vomiting ($P = 0.004$).

### Factors contributing to HRQL scores according to multivariate analyses

The patients’ characteristics that according to multivariate linear regression were predictive of the various EORTC QLQ-C30 symptom and functioning scores are presented in Table 4. Consistent with the results of simple comparisons, gender was predictive for physical functioning. The association between

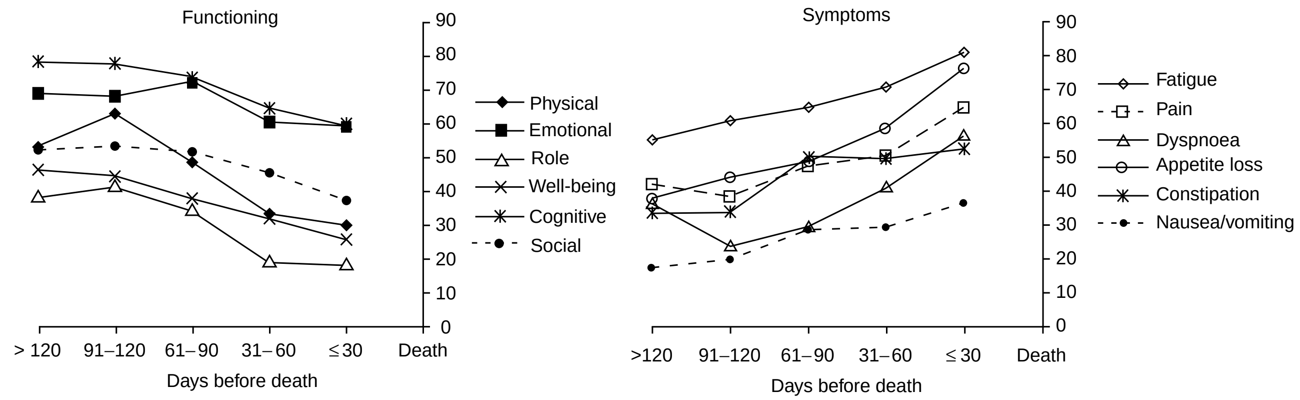

≤30 days, $n = 69$; 31–60 days, $n = 79$; 61–90 days, $n = 47$; 91–120 days, $n = 31$; >120 days, $n = 169$, each cohort representing different individuals.

**Figure 1** EORTC QLQ C30 scores from 5 cohorts of patients classified according to their survival after the assessment

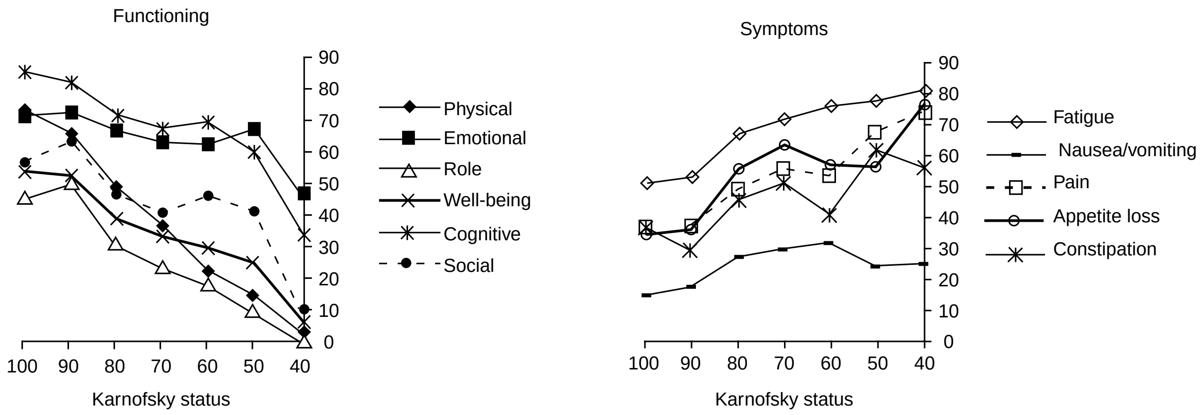

**Figure 2** EORTC QLQ C30 scores from 5 cohorts of patients, classified according to their Karnofsky performance status at the assessment point

 *© 2001 Cancer Research Campaign*

**Table 4** Linear regression models including sociodemographic and medical factors predictive of HRQL scores among advanced cancer patients

| Outcome variable | Factors contributing to outcome = model | Estimates for individual factors within model | | | Estimate for model[a] | Estimate for model when factor 'time from assessment to death' |
|---|---|---|---|---|---|---|
| | | Coef. | SE | *P*-value | R-square | was added R-square |
| Functioning scales | | | | | | |
| Physical[b] | Female sex | – 9.6 | 2.7 | < 0.001 | 0.30 | 0.35 |
| | Lung cancer | – 12.8 | 4.0 | 0.002 | | |
| | Karnofsky status > 70 | 30.5 | 2.7 | < 0.001 | | |
| Emotional | Age | 0.7 | 0.1 | < 0.001 | 0.10 | |
| | ≥ 13 years of education | 11.2 | 3.6 | 0.002 | | |
| | Karnofsky status > 70 | 11.3 | 2.7 | < 0.001 | | |
| Role[b] | Age | 0.5 | 0.1 | < 0.001 | 0.14 | 0.18 |
| | Karnofsky status > 70 | 24.0 | 3.1 | < 0.001 | | |
| General well-being[b,c] | ≤ 7 years of education[d] | 7.7 | 2.5 | 0.005 | 0.13 | 0.18 |
| | ≥ 13 years of education[d] | 9.9 | 3.7 | 0.007 | | |
| | Karnofsky status > 70 | 17.7 | 2.5 | < 0.001 | | |
| Cognitive[b] | Karnofsky status > 70 | 11.9 | 2.7 | < 0.001 | 0.05 | 0.09 |
| Social[b] | Age | 0.8 | 0.2 | < 0.001 | 0.14 | 0.15 |
| | Living with spouse | – 8.9 | 3.4 | 0.008 | | |
| | Karnofsky status > 70 | 20.0 | 3.3 | < 0.001 | | |
| Symptom scales/items | | | | | | |
| Fatigue[b] | Diagnoses 'other' | – 9.8 | 3.1 | 0.002 | 0.11 | 0.20 |
| | Karnofsky status > 70 | – 16.9 | 2.6 | < 0.001 | | |
| Nausea/vomiting[c] | Karnofsky status > 70[d] | – 8.9 | 3.1 | 0.004 | 0.02 | 0.05 |
| Pain[b] | Age | – 0.6 | 0.2 | < 0.001 | 0.13 | 0.15 |
| | Skeletal metastasis | 17.8 | 4.2 | < 0.001 | | |
| | Karnofsky status > 70 | – 17.4 | 3.7 | < 0.001 | | |
| | ≥ 13 years of education | – 14.1 | 4.8 | 0.004 | | |
| Diarrhoea | Gastrointestinal cancer | 13.0 | 3.4 | < 0.001 | 0.04 | |
| Dyspnoea[b] | Lung cancer | 31.2 | 5.6 | < 0.001 | 0.11 | 0.13 |
| | Cancer of breast/female genitals | 18.7 | 5.0 | < 0.001 | | |
| | Lung metastasis | 12.6 | 4.9 | 0.01 | | |
| Appetite loss[b] | Karnofsky status > 70 | – 16.9 | 4.0 | < 0.001 | 0.05 | 0.13 |
| Sleeping disturbances | Age | – 0.4 | 0.2 | 0.007 | 0.02 | |
| Constipation[b,c] | Ongoing radiotherapy | 19.4 | 6.2 | 0.002 | 0.09 | 0.11 |
| | Ongoing chemotherapy | 19.8 | 5.6 | < 0.001 | | |
| | Lung metastasis | – 15.1 | 5.3 | 0.005 | | |
| | Karnofsky status > 70[d] | – 12.9 | 3.9 | 0.001 | | |

[a]$P < 0.001$ for all models except for nausea/vomiting, $P = 0.004$ and for sleeping disturbances, $P = 0.007$.
[b]outcomes to which 'time from assessment to death' = 'closeness to death' contributed significantly in multivariate model.
[c]outcomes to which 'time from assessment to death' contributed significantly, but where this factor eliminated others from the multivariate model.
[d]factors that were eliminated from model when 'time from assessment to death' was added.

older age and better emotional and social functioning, lower pain scores and less sleeping disturbances was confirmed. Allowing for performance status, older age was also correlated to a better role functioning. The only impact of living with a spouse was worse social functioning scores (Table 4), whereas higher education was associated with less pain, better emotional functioning and greater general well-being. Except for physical functioning, neither diagnoses, location of metastases or ongoing cancer therapy were predictive of any functioning scores, whereas one or more of these factors contributed to most symptom ratings (Table 4).

Adding time from assessment to death to the models cited in Table 4, no factor was eliminated, except for level of education for general well-being and performance status for both nausea and vomiting and constipation (Table 4). Changes of the regression coefficient and p-value of individual factors were minor. However, time from assessment to death was found to make a significant contribution to all models (social functioning: $P = 0.006$, the others; $P < 0.001$) apart from those predicting emotional functioning, diarrhoea and sleeping disturbances. Hence, performance status and closeness to death were both significantly and independently associated with fatigue, pain, appetite loss and all functioning scales, except emotional functioning. The regression coefficient of the time variable within the models varied from –4.64 (pain) to –9.76 (appetite loss) for the symptom scores and from 3.61 (social) to 5.83 (physical) for the functioning scores. The contribution of variance from adding time from assessment to death is shown in Table 4.

Using ordered logistic regression, gender ($P = 0.006$) was included into the model for nausea and vomiting instead of Karnofsky status and was also not eliminated when allowing for time from assessment to death. For the model predicting constipation, having lung metastases was excluded. Otherwise identical results were achieved.

### DISCUSSION

The most striking result that emerges from the study, was the association between the HRQL ratings and performance status and closeness to death. Other clinical criteria such as diagnoses and location of metastasis contributed significantly to most symptom scores, whereas the overall influence of sociodemographic characteristics seemed less important.

Declining HRQL scores with physical deterioration and prior to death is consistent with the results of others (Morris et al, 1986; King, 1996; Axelsson and Sjoden, 1998). In this study, however, performance status and time from assessment to death were found to be independent contributors to most functioning scores. Although the finding does not contradict reports on HRQL scores being predictive of survival (Coates et al, 1997), it might be questioned whether such scores carry true prognostic information, or if they are mainly dependent on time of assessment due to an inevitable decline as death is approached. Anyway, our results confirm the necessity of taking differences in survival into account when comparing HRQL scores across settings and studies (Salisbury, 1999). Even in randomised studies of palliative care interventions, differences in HRQL scores might be related to differences in time from assessment to death rather than being a treatment effect, that is, if survival is not comparable among treatment groups.

As hypothesised and also reported by others (Klee et al, 1997; Michelson et al, 2000), older age was associated with better emotional functioning. In addition, age was predictive to role functioning, pain and sleeping disturbances, and opposed to results from a Norwegian population based survey (Hjermstad et al, 1998a), the impact of being old was merely positive. The observed differences between genders were consistent with normative EORTC QLQ-C30 data (Hjermstad et al, 1998a). In both samples, most differences were below clinical significance (less than 10). Compared to the larger population surveys, the present sample size was small, which could be the reason why in this study, most differences were also not statistically significant. The same explanation may be proposed for not detecting any overall positive impact of having a partner or higher education, as reported from general populations (Hjermstad et al, 1998a; Michelson et al, 2000). On the other hand, detailed analysis of population based data on self assessed health have also suggested that the contribution of such factors may be marginal when fine-grained arrays of medical information is taken into account (Moum, 1994). Our finding is, however, contradictory to the palliative care theory and experience, namely that living situation is of great importance to the patients' well-being. Further research on these issues seems necessary.

Surprisingly, being younger and living with a spouse were found to have a negative influence on social functioning. The explanation can be found in the wordings of the items within this scale. It is asked if physical condition or medical treatment has affected the respondents' family life and social activity. Patients, who are living alone or have low social activity in the first place, may be likely to answer 'not at all' and thus, obtain higher scores. Answering the questions also gives no indication whether a change is for the worse or for the better, hence these items do not seem to be an entirely useful measure of cancer patients' present social functioning.

The EORTC QLQ-C30 questionnaire was not developed for palliative care in particular and its appropriateness within these settings has been queried (Donelly and Walsh, 1996; Hearn and Higginson, 1997). However, the questionnaire has been widely used, rigorously developed, extensively tested, and its validity and reliability and responsiveness to changes have been established among advanced cancer patients (Kaasa et al, 1995; Cunningham et al, 1998). Although our results mainly confirm its ability to discriminate between groups of such patients, the floor effect that was observed on some functioning scale when Karnofsky status declined to 40, might be an indication that the questionnaire is not appropriated for those patients who are most severely ill.

Most of the regression models that were found to be predictive of the various HRQL scales explained only a minor part of the total variance. However, the coefficients of individual factors within the models were large, e.g. for physical functioning a decrease of ten in females compared to males was indicated, whereas Karnofsky status above 70 would give an increase of about 30. The multivariate analyses also confirmed the association between HRQL and the factors, for which significant group differences were found by simple comparisons. Consistent results were obtained by non-parametric statistics. Hence, we conclude that in studies of advanced cancer patients using HRQL outcomes, performance status, closeness to death, diagnoses and location of metastases as well as age and gender need consideration, either in design or in reporting of the findings. Living situation and education may be less relevant. However, the present study were based on data from a randomised trial evaluating a palliative care programme, and larger studies which are specifically designed for the purpose, may be needed to fully explore the impact of these and other social factors.

### ACKNOWLEDGEMENTS

The study was supported by grants from The Norwegian Cancer Society (grants no. 95147 and 98004), The Swedish Cancer Society (grants no. 3650-B9501-XAC) and The Norwegian Medical Association Fund for Quality Improvement (grants no. 4488/93). We are very grateful to all the patients who participated in the trial and willingly filled in the quality of life questionnaires. We would also like to thank Anne Lise Nessæther, Torgeir Fjermestad, Bjørg Petersvik, Nina Hassel and Frøydis Høyem Koteng for their indispensable participation in trial planning and organizing. The invaluable work of the research assistants Torbjørn Øvreness, Gro Underland and Bjørn Fougner and data manager Anders Skjeggestad is highly appreciated.

### REFERENCES

Aaronson NK, Ahmedzai SA, Bergman B, Bullinger M, Cull A, Duez NJ, Filiberti A, Flechtner H, Fleishman SB, de Haes JC, Kaasa S, Klee M, Osobo D, Razavi D, Rofe PB, Schraub S, Sneeuw K, Sullivan M and Takeda F (1993) for the European Organization for Research and Treatment of Cancer Study Group on Quality of Life. The EORTC QLQ-C30: A quality of life instrument for use in international clinical trials in oncology. *J Natl Cancer Inst* **85**: 365–376

Altman DG (1991) *Practical statistics for medical research* (1991) Chapman & Hall: London, pp 212–213

Axelsson B and Sjødén PO (1998) Quality of life of cancer patients and their spouses in palliative home care. Palliat Med 12: 29–39

Brazier JE, Harper R, Jones NMB, O'Cathain A, Thomas KJ, Usherwood T and Westlake L (1992) Validating the SF-36 health survey questionnaire: New outcome measures for primary care. *Br Med J* **305**: 160–164

Coates A, Porzsolt F and Osoba D (1997) Quality of life in oncology practice: prognostic value of EORTC QLQ-C30 scores in patients with advanced malignancy. *Eur J Cancer* **33**: 1025–1030

Cohen SR and Mount BM (1992) Quality of life in terminal illness: defining and measuring subjective well being in the dying. *J Palliat Care* **8**(3): 40–45

 *© 2001 Cancer Research Campaign*

Cunningham D, Pyrhönen S, James RD, Punt CJ, Hickish TF, Heikkila R, Johannesen TB, Starkhammar H, Topham CA, Awad L, Jacques C and Herait P (1998) Randomised trial of irinotecan plus supportive care versus supportive care alone after fluorouracil failure for patients with metastatic colorectal cancer. *Lancet* **352**: 1413–1418

Donelly S and Walsh D (1996) Quality of life assessment in advanced cancer. *Pall Med* **10**: 275–283

Fayers P, Aaronson N, Bjordal K and Sullivan M (1995) on behalf of EORTC Quality of Life Study Group. *EORTC QLQ-C30 Scoring Manual*. EORTC Study Group on Quality of Life, Brussels, Belgium

Hearn J and Higginson I (1997) Outcome measures in palliative care for advanced cancer patients: a review. *J Public Health Med* **19**: 193–199

Hearn J and Higginson I (1998) Do specialist palliative care teams improve outcomes for cancer patients? A systematic literature review. *Palliat Med* **12**: 317–332

Hjermstad MJ, Fayers PM, Bjordal K and Kaasa S (1998a) Health-related quality of life in the general Norwegian population assessed by the European Organization for Research And Treatment of Cancer Core Quality-of-Life Questionnaire: The QLQ = C30 (+3). *J Clin Oncol* **16**: 1188–1196

Hjermstad MJ, Fayers PM, Bjordal K and Kaasa S (1998b) Using reference data on quality of life -the importance of adjusting for age and gender, exemplified by the EORTC QLQ-C30 (+3). *Eur J Cancer* **34**: 1381–1389

Jenkinson C, Coulter A and Wright L (1993) Short form 36 (SF-36) health survey questionnaire: normative data for adults of working age. *Br Med J* **306**: 1437–1440

Jordhoy MS, Kaasa S, Fayers P, Øvreness T, Underland G and Ahlner-Elmqvist M (1999) Challenges in palliative care research; recruitment, attrition and compliance. Experience and recommendations from a prospective, randomised trial. *Palliat Med* **13**: 299–310

Jordhoy MS, Fayers P, Saltnes T, Ahlner-Elmqvist M, Jannert M and Kaasa S (2000) A palliative care intervention and death at home: a cluster randomised trial. *Lancet* **356**: 888–893

Kaasa S, Bjordal K, Aaronson N, Moum T, Wist E, Hagen S, Kvikstad A and The EORTC Study Group on Quality of Life (1995) The EORTC Core Quality of Life Questionnaire (QLQ-C30): Validity and reliability when analysed with patients treated with palliative radiotherapy. *Eur J Cancer* **31A**: 2260–2263

King MT (1996) The interpretation of scores from the EORTC Quality of Life questionnaire QLQ-C30. *Qual Life Res* **5**: 555–567

Klee M, Groenvold M and Machin D (1997) Quality of life of Danish women; Population-based norms for the EORTC QLQ-C30. *Qual Life Res* **6**: 27–34

Michelson H, Bolund C, Nilsson B and Brandberg Y (2000) Health-related quality of life measured by the EORTC QLQ-C30 — reference values from a large sample of Swedish population. *Acta Oncol* **39**: 477–484

Mor V, Allen S and Malin M (1994) The psychosocial impact of cancer on older versus younger patients and their families. *Cancer* **74**: 2118–2127

Morris JN, Suissa S, Sherwood S, Wright SM and Greer D (1986) Last Days: A study of the quality of life in terminally ill cancer patients. *J Chron Dis* **39**: 47–62

Moum T (1992) Self-assessed health among Norwegian adults. *Soc Sci Med* **35**: 935–947

Osoba D, Zee B, Pater J, Warr D, Kaizer L and Latreille J (1994) Psychometric properties and responsiveness of the EORTC Quality of life questionnaire (QLQ-C30) in patients with breast, ovarian and lung cancer. *Qual Life Res* **3**: 353–364

Osoba D, Rodrigues G, Myles J, Zee B and Pater J (1998) Interpreting the significance of changes in health related quality of life scores. *J Clin Onc* **16**: 139–144

Salisbury C (1999) Impact on quality of life. In *Providing a palliative care service. Towards an evidence base*, Bosanquet N, Salisbury C. (ed) pp 131–162. Oxford University Press: Oxford

Salisbury C, Bosanquet N, Wilkinson EK, Franks PJ, Lorentzon M and Naysmith A (1999) The impact of different models of specialist palliative care on patients' quality of life: a systematic literature review. *Palliat Med* **13**: 3–17

Smeenk FWJM, van Haastregt JCM, de Witte LP and Crebolder HFJM (1998) Effectiveness of home care programmes for patients with incurable cancer on their quality of life and time spent in hospital: a systematic review. *Br Med J* **316**: 1939–1944

SPSS Inc (1999) *SPSS (Statistical Package for the Social Sciences) for Windows: Base System User's Guide, release 9.0.1*. Chicago Illinois

Stata Corporation (1997) *Stata Statistical Software: Release 5.0*. Stata Press, College Station, Texas

Sullivan M, Karlson J and Ware JE (1994) Hasoenkat SF 36 *Svensk manual och tolkningsguide SF-36 Health survey. Swedish manual and interpretation guide*. Gothenburg, Sweden, Sahlgrenska Universitets Hospital

Wilkinson EK (1999) Problems of conducting research in palliative care. In *Providing a palliative care service. Towards an evidence base*, Bosanquet N, Salisbury C. (ed) pp 22–29. Oxford University Press: Oxford

*© 2001 Cancer Research Campaign*
